# Heteronemin Suppresses Lymphangiogenesis Through ARF-1 and MMP-9/VE-Cadherin/Vimentin

**DOI:** 10.3390/biomedicines9091109

**Published:** 2021-08-29

**Authors:** Hsien-Lin Chen, Yu-Chieh Su, Huang-Chi Chen, Jui-Hsin Su, Chang-Yi Wu, Shih-Wei Wang, In-Pin Lin, Chung-Yi Chen, Chien-Hsing Lee

**Affiliations:** 1 Division of General Surgery, Department of Surgery, Chi-Mei Medical Center, Liouying, Tainan 73657, Taiwan; ainchen72@gmail.com; 2 Department of Medicine, School of Medicine, I-Shou University, Kaohsiung 840203, Taiwan; hepatoma@gmail.com; 3 Division of Hematology-Oncology, Department of Internal Medicine, E-Da Hospital, Kaohsiung 824410, Taiwan; 4 Department of Internal Medicine, Kaohsiung Municipal Siaogang Hospital, Kaohsiung 81267, Taiwan; chenhuangchi@gmail.com; 5 Division of Pulmonary and Critical Care Medicine, Department of Internal Medicine, Kaohsiung Medical University Hospital, Kaohsiung 80708, Taiwan; 6 National Museum of Marine Biology & Aquarium, Institute of Marine Biotechnology, National Dong Hwa University, Pingtung 94401, Taiwan; x2219@nmmba.gov.tw; 7 Department of Biotechnology, Kaohsiung Medical University, Kaohsiung 80708, Taiwan; cywu@mail.nsysu.edu.tw; 8 Department of Biological Sciences, National Sun Yat-Sen University, Kaohsiung 804201, Taiwan; 9 Department of Medicine, Mackay Medical College, New Taipei City 252005, Taiwan; shihwei@mmc.edu.tw; 10 Graduate Institute of Natural Products, College of Pharmacy, Kaohsiung Medical University, Kaohsiung 80708, Taiwan; 11 Department of Pharmacology, College of Medicine, Kaohsiung Medical University, Kaohsiung 80708, Taiwan; inpin71126@msn.com; 12 Department of Nutrition and Health Science, School of Medical and Health Sciences, Fooyin University, Kaohsiung 83102, Taiwan; xx377@fy.edu.tw; 13 Department of Pharmacology, School of Post-Baccalaureate Medicine, College of Medicine, Kaohsiung Medical University, Kaohsiung 80708, Taiwan; 14 Department of Medical Research, Kaohsiung Medical University Hospital, Kaohsiung 80708, Taiwan; 15 Department of Biological Science and Technology, National Pingtung University of Science and Technology, Pingtung 91201, Taiwan

**Keywords:** lymphangiogenesis, heteronemin, zebrafish, ARF-1, MMP-9/VE-cadherin/vimentin, endothelial-to-mesenchymal transition

## Abstract

Lymphatic metastasis is a biological procedure associated with the pathogenesis of several diseases, especially in tumor metastasis. Therefore, regulation of lymphangiogenesis has become a promising strategy for cancer therapy. In this study, we aimed to investigate the anti-lymphangiogenic effect of heteronemin (SP-1) isolated from the sponge *Hyrtios* sp. in vitro and in vivo. Human lymphatic endothelial cells (LECs) were utilized to evaluate the anti-lymphangiogenic effect of SP-1 in vitro. Molecular docking, western blotting, flow-cytometry, MTT and ELISA were performed to investigate the mechanism of action. For in vivo approaches, the transgenic (fli1:EGFP; gata1:DsRed) zebrafish and mouse ear sponges were used. Molecular docking studies showed that SP-1 is a potent vascular endothelial growth factor receptor 3 (VEGFR-3)-binding compound. Treatment of LEC with SP-1 reduced the phosphorylation of VEGFR-3. SP-1 suppressed the development of the thoracic duct in zebrafish and mouse lymphangiogenesis ear sponges in vivo. Mechanistically, SP-1 induced the cell cycle arrest of LECs in the G0/G1 phase and reduced the downstream of VEGFR-3, such as phosphorylated MEK/ERK and NF-κB. In addition, SP-1 inhibited LECs’ tubulogenesis and migration through the ARF-1 and MMP-9/VE-cadherin/vimentin. Overall, anti-lymphangiogenic properties of SP-1 occur by downregulating the VEGFR-3 cascade, ARF-1 and MMP-9/VE-cadherin/vimentin. Collectively, these results proposed that SP-1 might be a potential candidate for the treatment of lymphangiogenesis-associated diseases.

## 1. Introduction

The lymphatic system, composed of lymph nodes, lymph ducts and lymphatic vessels, plays an important role in maintaining interstitial fluid homeostasis through intestinal water and macromolecules absorption and returning them back to the bloodstream [1]. Lymphangiogenesis, the generation of new lymphatic vessels from the preexisted ones, occurs during pathological conditions including in inflammation and cancer progression [2]. This process was regulated by vascular endothelial growth factors’ (VEGF) family members through binding to their receptors (VEGFRs), which are expressed at the surface of lymphatic endothelial cells (LECs) [3], especially in the VEGF-C/VEGFR-3 signaling pathway [4]. The activation of the VEGF-C/VEGFR-3 axis resulted in the phosphorylation on serine kinases, such as mitogen-activated protein kinase (MEK) and its downstream signaling extracellular signal-regulated kinase (ERK), to mediate the proliferation, tube formation, migration and survival of LECs [5]. In addition, activation of nuclear factor kappa B (NF-κB) up-regulates the VEGF-C/VEGFR-3 axis in LEC resulting in robust lymphangiogenesis [6]. Therefore, inhibition of lymphangiogenesis may be a potential strategy to reduce cancer growth and lymphatic metastasis.

Several studies have reported that endothelial cells (ECs) are involved in cancer progression through the endothelial-to-mesenchymal transition (EndoMT), a cellular process through which ECs are removed from their organized layer and migrate, occasionally intruding the surrounding connective tissue [7,8]. The EndoMT of ECs is a process to acquire a mesenchymal phenotype resulting in a loss of cell-cell junctions and endothelial markers, such as vascular endothelial (VE)-cadherin, and the gain of mesenchymal markers, such as matrix metallopeptidase 9 (MMP-9) and vimentin [9]. A recent study has also demonstrated that the EndoMT contributes to lymphangiogenesis [8]. Furthermore, the ADP-ribosylation factor (ARF) has demonstrated to mediate the migration of ECs resulting in the regulation of tumor lymphangiogenesis [10].

Heteronemin (SP-1) is a sesterterpene isolated from the sponge *Hyrtios* sp. and modulates the transcriptional level of several pathways, including cell cycle and apoptosis, and inflammation, against various cancer cells. SP-1 induces both the intrinsic and the extrinsic apoptotic pathways in human renal cell carcinoma [11] and chronic myeloid leukemia through the reduction in NF-κB [12]. In addition, SP-1 targeted NF-κB upstream protein mitogen-activated protein kinase (MAPK) and inhibited the phosphorylation of p38 and c-Jun NH2-terminal kinase (JNK) [11]. SP-1 suppressed the phosphoinositide 3-kinases (PI3K)/AKT pathway and the MAPK family member ERK in human renal cell carcinoma [11,12]. A recent study showed that SP-1 mediates the adhesion of the cell, angiogenic sprouting, transcription factors and vasculogenesis in cholangiocarcinoma cell lines [13]. Therefore, SP-1 seems to be a promising therapeutic compound to treat angiogenesis-related diseases or cancer.

In this study, a series of in vitro and in vivo experimental models was used to evaluate the anti-lymphangiogenic effects of SP-1. We demonstrated that SP-1 is a VEGFR-3 binding compound to reduce the phosphorylated VEGFR-3. In addition, it reduced the in vivo lymphangiogenic process of zebrafish and mouse ear sponges. Mechanistically, our results demonstrated that SP-1 possesses its anti-lymphangiogenic properties by the restraint of the downstream of VEGFR-3, such as MAPK (ERK and MEK) and NF-κB. In addition, SP-1 also inhibited LECs’ sprouting of new lymphatic vessels and migration associated with ARF1/MMP9/VE-cadherin/vimentin-mediated EndoMT. Overall, our data display novel pharmacological perspectives for using this compound in diseases related to abnormal lymphangiogenesis.

## 2. Materials and Methods

### 2.1. Materials and Reagents

Heteronemin is a sesterterpene isolated from the sponge *Hyrtios* sp., as previously described [14]. Dimethylsulfoxide (DMSO), 3-[4,5-Dimethylthiazol-2-yl]-2,5-diphenyltetrazolium bromide (MTT) and other chemical agents were obtained from Sigma-Aldrich (St. Louis, MO, USA). Rabbit monoclonal antibodies specific for ARF-1, lymphatic vessel endothelial receptor-1 (LYVE-1), NF-κB, prospero homeobox 1 (PROX-1) and VE-cadherin were purchased from the Proteintech Group (Rosemont, IL, USA). Phospho-ERK (Thr202/Tyr204), phospho-MEK (Ser217/221), phospho-p65 (Ser536), ERK, MEK and β-actin were purchased from Cell Signaling Technologies (Boston, MA, USA). MMP-9 was purchased from Santa Cruz Biotechnology (Santa Cruz, CA, USA). The rabbit polyclonal antibody specific for VEGFR-3 and phospho-VEGFR-3 (Tyr1230/1231) was purchased from Cell Application (San Diego, CA, USA). Matrigel was obtained from BD Biosciences (Bedford, MA, USA).

### 2.2. Molecular Docking

The structure of SP-1 and VEGFR-3 proteins was constructed by docking optimization (CDOCKER) and optimized with the CHARMm force field using Discovery Studio 4.1 (DS) (BIOVIA, San Diego, CA, USA). The receptor structure of VEGFR-3 was obtained from the Protein Data Bank (PDB; Accession: 4BSJ_A) and drawn using ChemDraw Ultra 9.0. The binding of SP-1 and VEGFR-3 with the most suitable energy was estimated with -CDOCKER (−kcal/mol).

### 2.3. Western Blot Analysis

After the treatment with the indicated concentration of SP-1 in human LECs, cells were terminated by the addition of a lysis buffer containing a protease inhibitor cocktail (Roche, Mannheim, Germany). Total cell lysates were electrophoresed using SDS-PAGE and subsequently transferred to polyvinylidene difluoride membranes. After blocking the blots with 4% bovine serum albumin, they were treated with a primary antibody and then a peroxidase-conjugated secondary antibody. The blots were visualized using enhanced chemiluminescence and monitored using the UVP Biospectrum system (UVP, Upland, CA, USA).

### 2.4. Enzyme Linked Immunosorbent Assay

Phospho-VEGFR-3 was measured by using an enzyme linked immunosorbent assay (ELISA) kit (RayBiotech, Norcross, GA, USA) according to the manufacturer’s instructions.

### 2.5. Thoracic Duct Formation in Zebrafish

The transgenic (fli1:EGFP; gata1:DsRed) zebrafish, which expresses EGFP at endothelial cells and DsRed at blood cells [15], was kindly provided by Prof. Chang-Yi Wu (National Sun Yat-Sen University, Kaohsiung, Taiwan). The sampling procedures in zebrafish were overseen by the Institutional Animal Care and Use Committee (Protocol 104077) at Kaohsiung Medical University, Taiwan. Transgenic (fli1:EGFP; gata1:DsRed) zebrafish embryos at 24 hpf were treated with the indicated concentrations of SP-1. After treatment, embryos were anaesthetized and observed under an inverted fluorescence microscope (Nikon Eclipse TE2000-U, Tokyo, Japan) to evaluate the thoracic duct. Quantification of defective thoracic duct formation was calculated as previously described [16]. A total of 50 embryos were evaluated for each experimental condition.

### 2.6. Mouse Ear Sponge Assay

All animal experiments were performed following the National Institutes of Health Guidelines for Animal Research (Guide for the Care and Use of Laboratory Animals) and were approved by the Kaohsiung Medical University Institutional Animal Care and Use Committee (Protocol 104077). The mouse (male B6 mice of 6 weeks) ear sponge assay was performed following previously described work [16]. In brief, sterile gelatin sponges were cut into small pieces of implants (~3 mm^3^) and treated with serum-free Dulbecco’s modified Eagle medium containing recombinant VEGF-C (1 μg/mL) and with or without indicated concentrations of SP-1. Then, they were embedded in interstitial type I collagen gel to implant to the mouse ears. SP-1 was given to the implant site behind the ear three times a week for one month and then sacrificed. At the time of sacrifice, the ears were taken and analyzed by immunostaining with LYVE-1 (1/200; R&D, Abingdon, UK).

### 2.7. Cell Cytotoxicity Assay

The human LECs were purchased from PromoCell (Heidelberg, Germany). LECs were cultured in EGM-2MV medium consisting of EBM-2 basal medium and the SingleQuots kit (Lonza). The 5 × 10^3^ LECs cells were seeded onto 96-well plates and incubated with EGM-2MV medium in the presence of a vehicle (DMSO) or SP-1 (0.1–100 μM) for 24 h. Cell viability was determined by MTT assay. Cells were treated with 0.5 mg/mL MTT for 4 h in 37 °C, and then the formazan crystals in the cells were solved with 100 μL DMSO. Absorbance was read at 560 nm using a microplate reader.

The 5 × 10^3^ LECs cells were seeded onto 96-well plates and incubated with EGM-2MV medium in the presence of a vehicle (DMSO) or the indicated concentration of SP-1 for 8 h. Release of lactate dehydrogenase (LDH) into the medium was measured using a cytotoxicity assay kit (Promega, Madison, WI, USA) according to an established protocol [17].

### 2.8. Cell Cycle Analysis

Cells were seeded at a density of 5 × 10^5^ cells/mL medium per well in 6-well plates and were cultured overnight. Cells were then treated with various concentrations of SP-1, after which they were harvested and stained with a PI staining kit (BD Biosciences, San Jose, CA, USA). Samples were evaluated by flow cytometry (FACS Calibur; Becton Dickinson, Mountain View, CA, USA) and analyzed using FlowJo software (Tree Star, Ashland, OR, USA). Etoposide was used as a positive control.

### 2.9. Capillary Tube Formation Assay

LECs were seeded at a density of 1.6 × 10^4^ cells per well of a Matrigel-coated 96-well plates and incubated in EGM-2MV medium and the indicated concentration of SP-1. The detection and quantification of LECs’ tube formation were examined according to previously described procedures [17].

### 2.10. Cell Migration Assay

The cell migration assay was performed in Transwell inserts of an 8 μm pore size (Corning, NY, USA). LECs (2.5 × 10^4^/well) were seeded onto the upper chamber with EBM-2 basal medium. The upper chamber was placed and incubated in the bottom chamber with EGM-2MV medium containing the indicated concentration of SP-1 for 8 h. Cell migration was determined based on our previous study [17].

### 2.11. Statistical Analysis

Data points represent the mean ± standard error of mean (SEM) in at least triplicate experiments. Statistical analyses of data were conducted with one-way ANOVA followed by Tukey’s post-hoc test. The difference is significant if the *p* value is <0.05.

## 3. Results

### 3.1. Heteronemin Is a VEGFR-3 Binding Compound in Human Lymphatic Endothelial Cells

**Figure 1:**
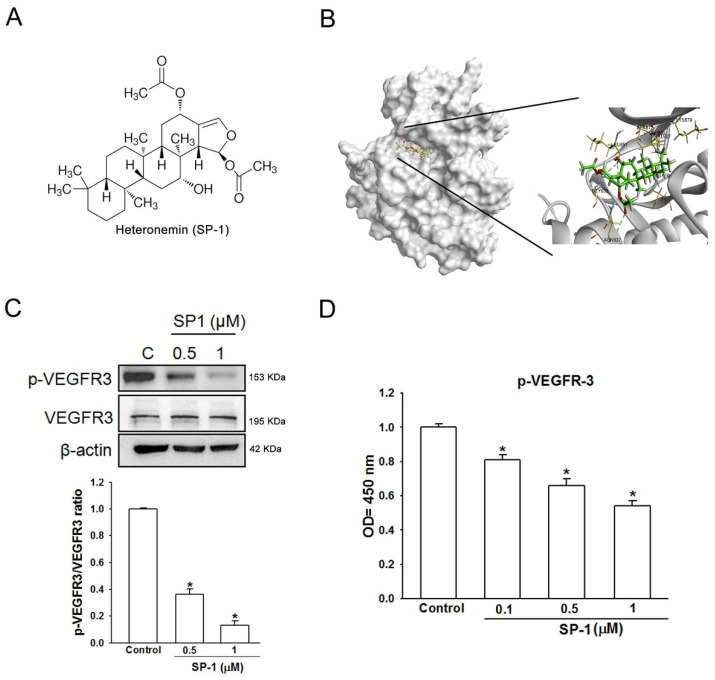
Heteronemin is a VEGFR-3 binding compound in human lymphatic endothelial cells. (**A**) The structure of heteronemin (indicated as SP-1). (**B**) Docking models of SP-1-targeted VEGFR-3. The structure of VEGFR-3 was downloaded from PDB (accession: 4BSJ_A) and represented as gray. SP-1, drawn by ChemDraw Ultra 9.0, rendered in the representation of green stick. Close-up of SP-1 docking site (best energy mode) was prepared using Discovery Studio 4.1. (**C**) Human lymphatic endothelial cells (LECs) were treated with SP-1. Then, the expression of VEGFR-3 and phospho-VEGFR-3 (p-VEGFR-3) was determined by Western blot analysis. The quantitative densitometry of the relative levels of VEGFR-3 and phospho-VEGFR-3 was measured by Image-Pro Plus. (**D**) Cells were stimulated with SP-1; the phospho-VEGFR-3 protein expression in cell lysate was measured by ELISA. A representation experiment is shown as the mean ± S.D. for three wells (* *p* < 0.05). Similar results were observed from three independent experiments.

Vascular endothelial growth factor receptor 3 (VEGFR-3) has shown to be involved in tumor-associated lymphangiogenesis and lymphatic metastasis [18]. To determine whether heteronemin (SP-1) binds the VEGFR-3, the molecular docking of SP-1 with VEGFR-3 was performed. The SP-1-VEGFR-3 binding modes were produced in receptor cavities with ten poses according to CDOCKER and the CHARMm force field. The binding of SP-1 and VEGFR-3 with the best energy was evaluated with -CDOCKER (−464.432 kcal/mol). SP-1 was proposed to interact with the V859, A877, K879, V910, V927, N937 or C1054 residues of VEGFR-3 (Figure 1B), suggesting that SP-1 may preferably and specifically associate with this proposed “pocket” of VEGFR-3. Western blot and ELSIA analysis demonstrated that phosphorylation of VEGFR-3 was decreased by treatment with SP-1 (Figure 1C,D). These results suggest that SP-1 was bound to VEGFR-3 to alleviate its phosphorylation.

### 3.2. Heteronemin Inhibits Zebrafish and Mouse Lymphangiogenesis In Vivo

**Figure 2:**
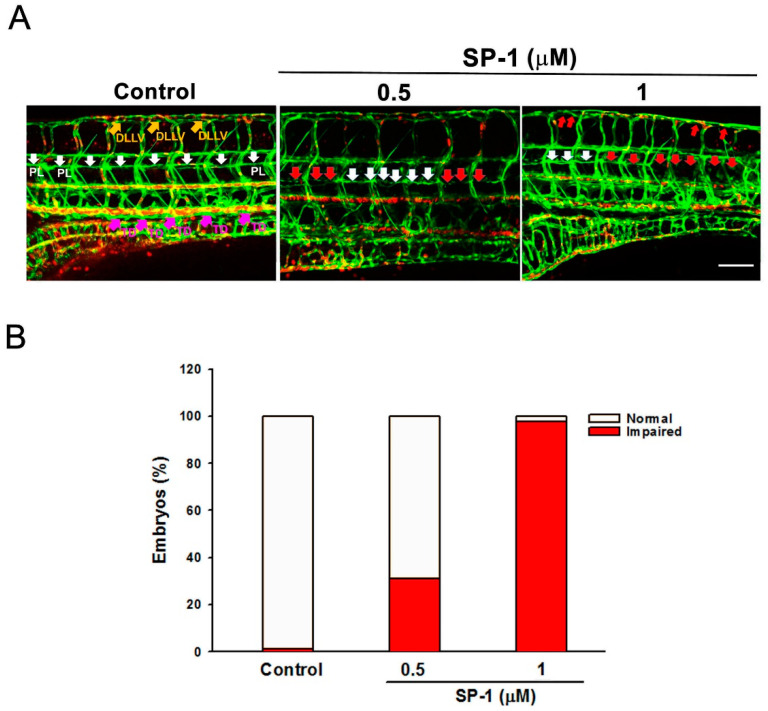
Heteronemin blocks thoracic duct development in zebrafish model. Transgenic (fli1:EGFP;gata1:DsRed) zebrafish embryos were treated with the indicated concentrations of heteronemin (represented as SP-1), and then thoracic duct length was analyzed via fluorescence microscopy. DLLV indicated as dorsal longitudinal lymphatic vessel (orange arrows); PL, parachordal LEC (white arrows) and TD, thoracic duct (pink arrows). (**A**) Representative pictures of thoracic duct of zebrafish treated with indicated concentration of SP-1 (bar = 100 μm). (**B**) Quantification of the defective formation of thoracic duct at 5 dpf determined by the percentages of embryos. A total of 50 embryos were analyzed in each experimental condition.

In zebrafish, the generation of the thoracic duct, located between the dorsal aorta and the posterior cardinal vein, is to study the model of lymphangiogenesis [17]. Embryos from a transgenic (fli1:EGFP; gata1:DsRed), driving GFP expression in the endothelium and DsRed in blood cells, were incubated with 0.5 and 1 μM SP-1. After 4 days, we analyzed the morphology of the fish through the observation of thoracic duct formation. SP-1 at 0.5 μM produced a significant defection in the thoracic duct of zebrafish compared to the control group (Figure 2A). The thoracic ducts are impaired in 90% of 1 μM SP-1-treated zebrafishes, and only lower than 10% of embryos had a normal-length thoracic duct (Figure 2B).

**Figure 3:**
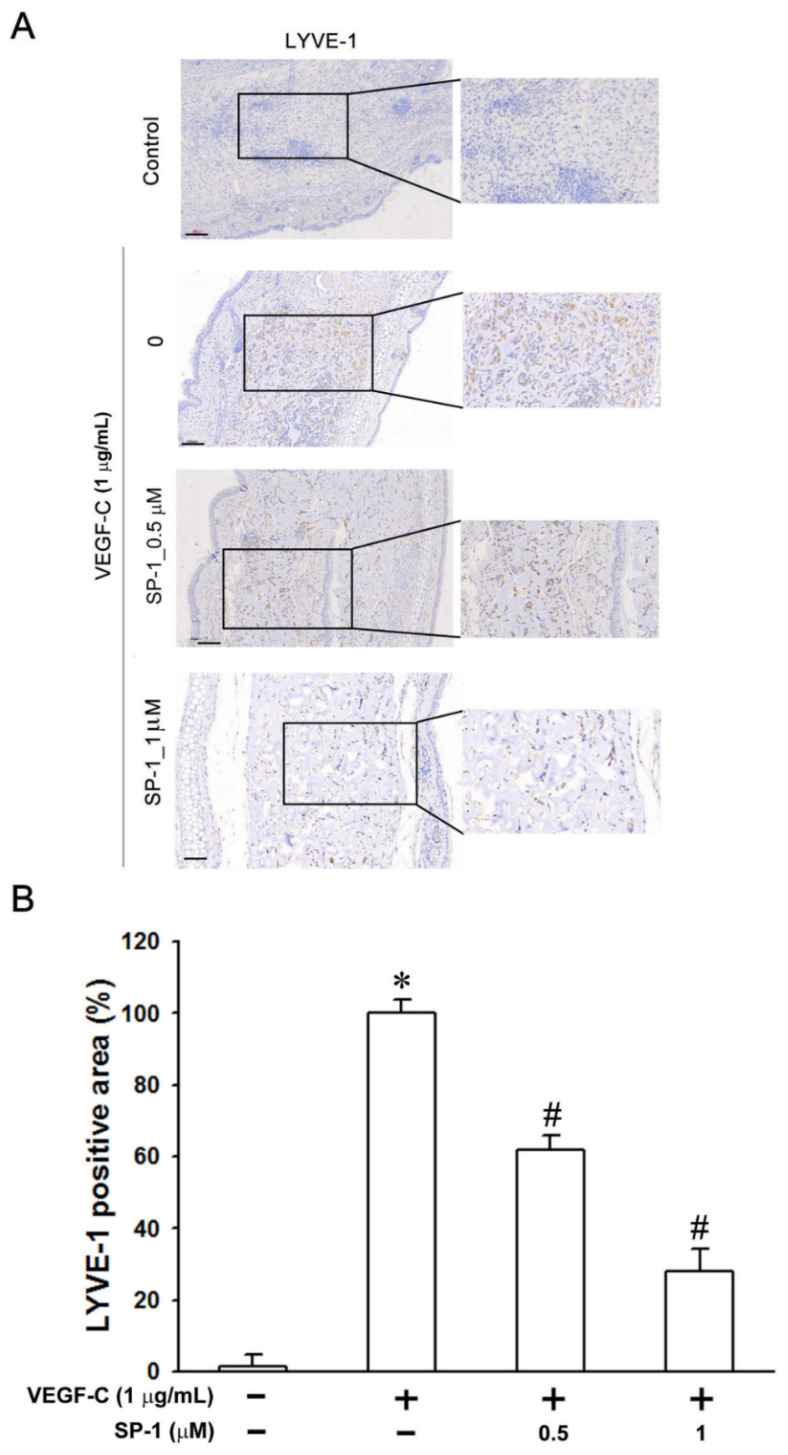
Heteronemin impairs the in vivo VEGF-C-stimulated lymphangiogenesis in mouse ear collagen sponges. Gelatin sponges were soaked with either medium containing VEGF-C (1 μg/mL) as a positive control or VEGF-C combined with the indicated concentration of heteronemin (represent as ‘SP-1’ in the graphs). Sponges were implanted between the two skin layers of mice ears for 3 weeks. (**A**) Lymphatic vasculatures were examined by LYVE-1 immunostainings. (bars = 100 and 70 μmon higher magnification). (**B**) The graphs represent the computerized quantification of the densities of lymphatic vessels, defined as the area occupied by vessels divided by the area of the sponge section. The data are expressed as the means ± SEM of five mice. * *p* < 0.05 versus medium sponges; # *p* < 0.05 versus VEGF-C-stimulated sponges.

In addition, the mouse ear sponge assay has also been reported in the investigation of lymphangiogenesis [19]. A higher level of lymphangiogenesis was observed in sponges soaked with VEGF-C (1 μg/mL) compared to the control (Figure 3). The in vivo treatment with SP-1 (0.5 and 1 μM) reduced VEGF-C stimulated lymphangiogenesis (Figure 3). Based on the above results, SP-1 was demonstrated to reduce stimulated lymphangiogenesis in vivo.

### 3.3. Heteronemin Induces G0/G1 Arrest in Human Lymphatic Endothelial Cells

**Figure 4:**
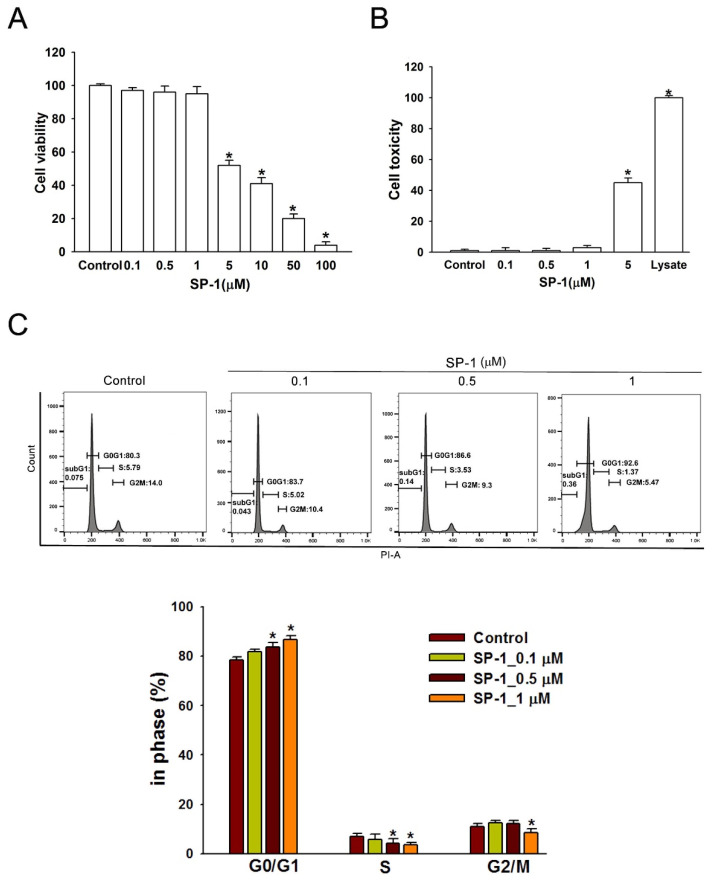
Heteronemin inhibited cell growth and arrested the distribution of cell cycle in human lymphatic endothelial cells. (**A**) LECs were incubated with various doses (0–100 μM) of heteronemin (indicated as SP-1) in a 96-well plate for 24 h. Cell viability was determined by MTT assay after treatment. The maximal non-toxic dose was chosen for further experiments. (**B**) Cells were treated with SP-1 for 8 h; then, the cytotoxicity was determined using LDH assay. (**C**) Cells were treated with various concentrations of SP-1 for 24 h and were then analyzed using flow cytometry. Histograms represent the percentage of cells in each cell cycle phase. The graph corresponds to the distribution of cell subpopulation percentages expressed as means ± SEM of five independent assays. * *p* < 0.05 compared with solvent control (0.01% DMSO).

Human LECs were exposed to various concentrations of SP-1 (0–100 μM) for 24 h, and then a MTT assay was performed to determine cell viability. Figure 4A shows that SP-1 at a concentration over 5 μM was toxic in human LECs compared with the control group. Moreover, SP-1-induced cell cytotoxicity of human LECs was confirmed using the lactate dehydrogenase (LDH) assay. SP-1 did not increase LDH release in human LECs at a lower concentration (<1 μM) (Figure 4B).

To investigate the major underlying mechanisms of SP-1-induced growth inhibition of LECs, we next analyzed SP-1 on the cell cycle distribution of LECs using flow cytometry. As shown in Figure 4C, the percentages of cells in the G0/G1 phase were significantly increased after exposure to SP-1. However, the percentage of cells in the S- and G2/M-phase was significantly reduced in SP-1-treated LECs, suggesting SP-1 induces G0/G1 arrest in human LECs.

### 3.4. Heteronemin Suppresses MEK/ERK and NF-κB Downstream Activation in Human Lymphatic Endothelial Cells

**Figure 5:**
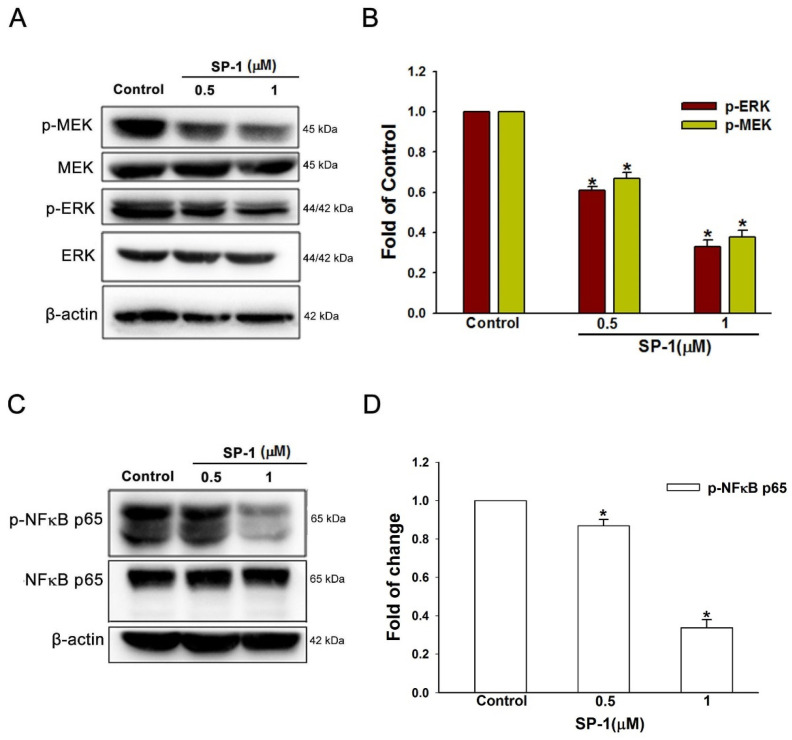
Heteronemin inhibited mitogen-activated protein kinase and transcription factors in human lymphatic endothelial cells. Cells were treated with the indicated concentrations of heteronemin (represented as SP-1); subsequently, the indicated (**A**) MEK/ERK and (**C**) p65 phosphorylated and total proteins were determined by Western blot analysis. The quantitative densitometry of phosphorylated (**B**) MEK/ERK and (**D**) p65 protein was performed with Image-Pro Plus. Data are expressed as mean ± SEM of five independent experiments. * *p* < 0.05, compared with the control group.

A mechanistic exploration of the anti-lymphangiogenesis effect of SP-1 in human LECs was next conducted by focusing on the downstream of the VEGF-C/VEGFR-3 axis, such as MEK/ERK and NF-κB [15,20]. MEK and its downstream signaling ERK have been implicated in cancer progression processes such as metastasis, angiogenesis or lymphangiogenesis [21,22]. We therefore examined whether the MEK/ERK pathway is involved in SP-1-mediated lymphangiogenesis. Treatment with SP-1 decreased MEK and ERK phosphorylation (Figure 5A,B), suggesting that SP-1 acts through the MEK/ERK pathway to diminish the lymphangiogenesis in human LECs.

NF-κB activation up-regulates the VEGF-C/VEGFR-3 axis to promote lymphangiogenesis and lymph node metastasis [23,24]. Treatment with SP-1 was found that the reduction in NF-κB p65 phosphorylation (Figure 5C,D). Taken together, these results demonstrated that the anti-lymphangiogenic effects of SP-1 are associated with MEK/ERK and NF-κB.

### 3.5. Heteronemin Suppresses Tube Formation and Migration through ARF1 and MMP9/VE-Cadherin/Vimentin-Mediated Endothelial-Mesenchymal Transition in Human Lymphatic Endothelial Cells

**Figure 6:**
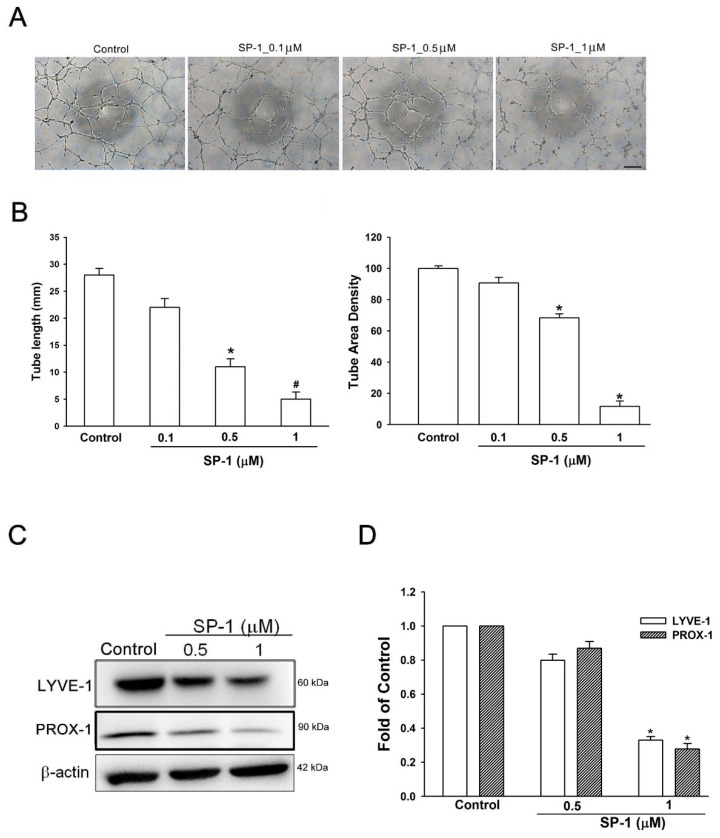
Heteronemin suppressed the tube formation, migration and phosphorylation of VEGF-C/VEGFR-3/LYVE-1 in human lymphatic endothelial cells. (**A**) Cells were treated with the indicated concentrations of heteronemin (indicated as SP-1). The capillary-like structure formation was examined by tube formation (scale bar = 500 μm; 20× magnification). (**B**) The quantification of tube formation was performed using Image-J to validate the anti-lymphangiogenic property of SP-1. (**C**) LECs were treated with SP-1. Then, the expression of LYVE-1and PROX-1 was determined by Western blot analysis. (**D**) The quantitative densitometry of the relative levels of LYVE-1 and PROX-1 was measured by Image-Pro Plus. Data are expressed as the mean ± SEM of at least three independent experiments. * *p* < 0.05; ^#^ *p* < 0.01 compared with the control group.

Figure 6A revealed that the tubular network was disorganized after exposure to SP-1 for 8 h. SP-1 dose-dependently suppressed lymphatic tubulogenesis (Figure 6B). Furthermore, administration of SP-1 induced a decrease in the expression of LYVE-1 and PROX-1, which are LEC markers (Figure 6C,D). These results indicate the suppression tube formation of human LECs by SP-1.

**Figure 7:**
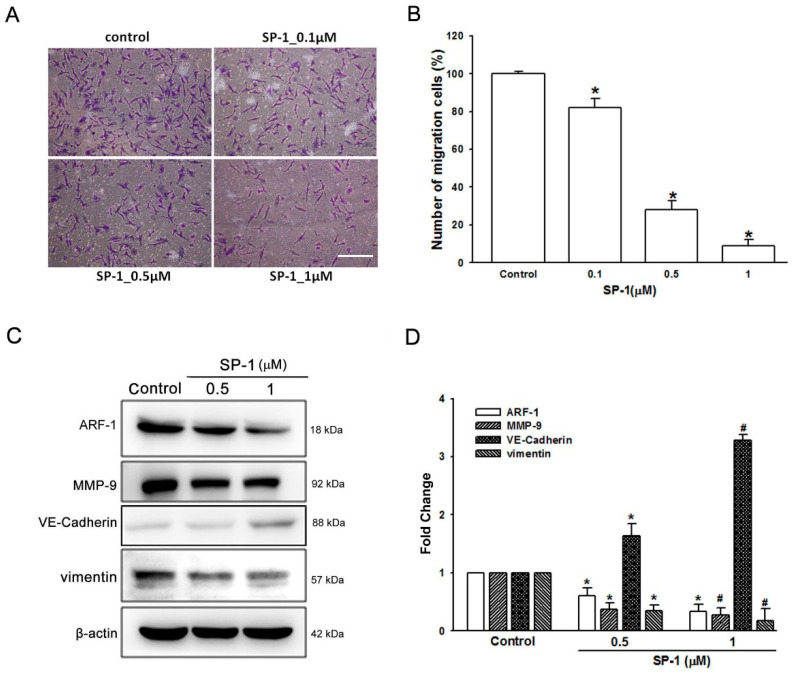
Heteronemin suppressed the migration and endothelial-to-mesenchymal transition of human lymphatic endothelial cells. Cells were treated with the indicated concentrations of heteronemin (represented as SP-1). (**A**) Cell migration was examined by Transwell assays (scale bar=100 µm; magnification ×100). (**B**) The quantification of migration was performed using Image-J to validate the anti-lymphangiogenic function of SP-1. (**C**) LECs were treated with SP-1. Then, the expression of ARF-1 and endothelial-mesenchymal transition-related proteins (MMP-9, VE-cadherin and vimentin) was determined by Western blot analysis. (**D**) The quantitative densitometry of the relative levels of ARF-1, MMP-9, VE-cadherin and vimentin was measured by Image-Pro Plus. Data are expressed as the mean ± SEM of at least three independent experiments. * *p* < 0.05, ^#^ *p* < 0.01 compared with the control group.

The migration of LECs is a characteristic in the process of lymphangiogenesis; the transwell migration assay was performed to evaluate the influence of SP-1 on the migration of LECs [17]. We found that SP-1 significantly diminishes the numbers of human LECs in a dose dependent manner (Figure 7A,B). The ARF family has been demonstrated to mediate several functions, including in cytoskeleton remodeling, cell cycle distribution, cell migration and adhesion [25]. To investigate whether SP-1 inhibits the migration of LECs through the regulation of ARF, we examined the expression of ARF-1 after treatment with SP-1 by chemiluminescence detection. In our experiments, we observed that the expression of ARF-1 was downregulated in SP-1-treated human LECs (Figure 7C,D). In addition, EndoMT plays an important role in tumor invasion and increases motility correlated with lymphangiogenesis [8]. During EndoMT, the reduction of endothelial cell markers such as VE-cadherin occurs, whereas the induction of mesenchymal markers such as MMP-9 and vimentin occurs [26]. Here, results from this study demonstrated a strong increase in VE-cadherin, whereas a decrease in MMP-9 and vimentin in SP-1-treated human LECs was observed (Figure 7C,D). Collectively, these findings suggest that ARF-1 and EndoMT contribute to the suppression effect of SP-1 in the migration ability of human LECs.

## 4. Discussion

**Figure 8:**
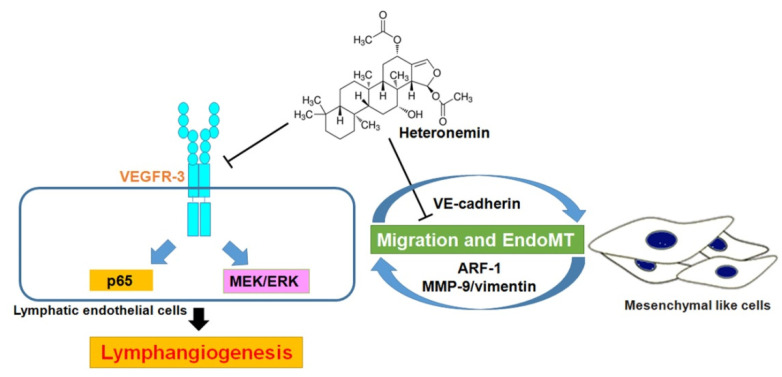
Schema of heteronemin-induced anti-lymphangiogenic mechanism in human lymphatic endothelial cells. This study reveals heteronemin as a promising anti-lymphangiogenic agent. Heteronemin may possess anti-lymphangiogenesis effect by reducing VEGFR-3 and its downstream NF-κB p65 and MEK/ERK signaling pathways and ARF-1, as well as the induction of VE-cadherin and the reduction in MMP-9 and vimentin, which is associated with EndoMT, in human lymphatic endothelial cells. T-shaped lines indicate inhibition.

Lymphatic vessels mediate the drainage of fluid, transport of lipid and inflammation. Hence, excessive lymphangiogenesis induces cancer cell metastasis, inflammation and graft (renal and corneal) rejection, and blocking lymphangiogenesis has potential as a treatment for different pathological conditions [27]. In the present study, we provide in vitro and in vivo evidence that heteronemin (SP-1; isolated from the sponge *Hyrtios* sp.) is a potent inhibitor of lymphangiogenesis. Mechanistically, this anti-lymphangiogenic effect depends on the abolishment of VEGFR-3 phosphorylation and the downstream signaling pathway, as well as ARF-1/MMP-9/VE-cadherin/vimentin-mediated EndoMT (Figure 8).

Several studies have demonstrated that the MEK/ERK pathway is considered as the crucial regulator in several cell functions, such as growth, proliferation, angiogenesis and lymphangiogenesis [22,28]. The MEK/ERK pathway has been indicated to take part in lymphangiogenesis. Adrenomedullin was involved in lymphangiogenesis and promoted LEC proliferation through the MEK/ERK pathway [29]. The MEK/ERK is an important downstream pathway for VEGF-C/VEGFR-3 signaling in the regulation of LEC migration and tumor-induced lymphangiogenesis [30]. The brain-derived neurotrophic factor facilitated VEGF-C/VEGFR-3-dependent lymphangiogenesis through the MEK/ERK signaling pathway [28]. Here, results from this study demonstrated that treatment with SP-1 decreased the phosphorylation of MEK and ERK, which indicates that the MEK/ERK has a key role in SP-1-reduced VEGFR-3-dependent lymphangiogenesis. Thus, our results show that SP-1 restrains VEGFR-3 expression and lymphangiogenesis in LECs via the MEK/ERK signaling pathway.

ARF-1 has been indicated to play a role in the mediation of VEGF signaling through ARF1 deactivation, leading to a significant decrease in VEGF secretion [31]. The downregulation of ARF-1 disrupted VEGF-induced vascular permeability and capillary tubule formation [28]. The ARF-1 inhibitor can induce the regression of alkali-induced corneal neovascularization through increased endothelial cell apoptosis and downregulated intracorneal VEGF expression [32]. In addition, ARF-6 exited LECs to regulate lymphangiogenesis through mediating cell migration [10]. Our findings showed that the expression of ARF-1 was downregulated in SP-1-treated human LECs, suggesting that ARF-1 is involved in the anti-lymphangiogenesis effects of SP-1. Furthermore, EndoMT was indicated to influence capacities of LECs including in migration, tube formation and lymphangiogenesis [8]. EndoMT serves as a process with the loss of endothelial phenotypes and the gain of mesenchymal phenotypes, as well as MMPs in cancer [33]. A recent study demonstrated that SP-1-induced MMP remodeling and cell adhesion resulted in vasculogenesis in cholangiocarcinoma [13]. These factors are associated with EndoMT. However, the functions of SP-1 on the migration and EndoMT of LECs still lacked investigation. In the present study, we first found that EndoMT-related proteins were mediated in SP-1-treated human LECs, indicating that SP-1 induced the inhibition of lymphangiogenesis associated with EndoMT. The following mechanistic dimension revealed that the migration and the EndoMT formation of LECs were impaired by the elevated VE-cadherin and reduction in MMP-9 and vimentin under SP-1 treatment. All these results clearly demonstrated that SP-1 could mediate MMP-9, VE-cadherin and vimentin to suppress the EndoMT and migration of LECs, which thus decelerated the lymphatic vessel formation.

Nemours marine drugs demonstrate lymphangiogenic inhibitory properties and regulate the VEGFR-3 signaling pathway, such as phomaketide A isolated from the marine endophytic fungal strain *Phoma* sp. NTOU4195 [17], tuberazines C obtained from the ethanolic extract of Taiwanese zoanthid *Palythoa tuberculosa*. [34], toluquinol isolated from the culture broth of the marine fungus *Penicillium* sp. HL-85-ALS5-R004 [16], and fucoidan derived from *Undaria pinnatifida* sporophylls [35]. In the present study, SP-1 is a sesterterpene isolated from the sponge *Hyrtios* sp. Thus, it has been experimentally demonstrated that the marine environment is a very rich source for the discovery of anti-lymphangiogenic drugs. In addition, SP-1 has also been indicated to possess anti-cancer activity against various cancers [13,36,37,38,39,40,41,42,43]. Meanwhile, lymphangiogenesis enhances the metastatic outgrowth of cancer. In this context, SP-1 may have a promising therapeutic value in tumor-associated lymphangiogenesis. In addition, lymphangiogenesis occurs during the fibrosis production in several pathologic conditions [44,45]. The effect of SP-1 in fibrosis-related lymphangiogenesis should be an interesting subject for further investigations.

## 5. Conclusions

In conclusion, the data presented here bring new insights into the anti-lymphangiogenic properties of heteronemin by downregulating the VEGFR-3 cascade, ARF-1, MMP-9/VE-cadherin/vimentin-mediated EndoMT. The present work emphasizes the potential value of heteronemin in the pharmacological treatment of pathologies with excessive lymphangiogenesis due to the suppression of LECs’ migration and tubulogenesis.

## Data Availability

Not applicable.
