# Correction: Chen et al. Heteronemin Suppresses Lymphangiogenesis Through ARF-1 and MMP-9/VE-Cadherin/Vimentin. *Biomedicines* 2021, *9*, 1109

**DOI:** 10.3390/biomedicines12112609

**Published:** 2024-11-15

**Authors:** Hsien-Lin Chen, Yu-Chieh Su, Huang-Chi Chen, Jui-Hsin Su, Chang-Yi Wu, Shih-Wei Wang, In-Pin Lin, Chung-Yi Chen, Chien-Hsing Lee

**Affiliations:** 1 Division of General Surgery, Department of Surgery, Chi-Mei Medical Center, Liouying, Tainan 73657, Taiwan; ainchen72@gmail.com; 2 Department of Medicine, School of Medicine, I-Shou University, Kaohsiung 840203, Taiwan; hepatoma@gmail.com; 3 Division of Hematology-Oncology, Department of Internal Medicine, E-Da Hospital, Kaohsiung 824410, Taiwan; 4 Department of Internal Medicine, Kaohsiung Municipal Siaogang Hospital, Kaohsiung 81267, Taiwan; chenhuangchi@gmail.com; 5 Division of Pulmonary and Critical Care Medicine, Department of Internal Medicine, Kaohsiung Medical University Hospital, Kaohsiung 80708, Taiwan; 6 National Museum of Marine Biology & Aquarium, Institute of Marine Biotechnology, National Dong Hwa University, Pingtung 94401, Taiwan; x2219@nmmba.gov.tw; 7 Department of Biotechnology, Kaohsiung Medical University, Kaohsiung 80708, Taiwan; cywu@mail.nsysu.edu.tw; 8 Department of Biological Sciences, National Sun Yat-Sen University, Kaohsiung 804201, Taiwan; 9 Department of Medicine, Mackay Medical College, New Taipei City 252005, Taiwan; shihwei@mmc.edu.tw; 10 Graduate Institute of Natural Products, College of Pharmacy, Kaohsiung Medical University, Kaohsiung 80708, Taiwan; 11 Department of Pharmacology, College of Medicine, Kaohsiung Medical University, Kaohsiung 80708, Taiwan; inpin71126@msn.com; 12 Department of Nutrition and Health Science, School of Medical and Health Sciences, Fooyin University, Kaohsiung 83102, Taiwan; xx377@fy.edu.tw; 13 Department of Pharmacology, School of Post-Baccalaureate Medicine, College of Medicine, Kaohsiung Medical University, Kaohsiung 80708, Taiwan; 14 Department of Medical Research, Kaohsiung Medical University Hospital, Kaohsiung 80708, Taiwan; 15 Department of Biological Science and Technology, National Pingtung University of Science and Technology, Pingtung 91201, Taiwan

**Error in Affiliation**

The affiliation number 1, Division of General Surgery, Department of Surgery, Liuying Chi-Mei Medical, Tainan 73657, Taiwan changed its information to Division of General Surgery, Department of Surgery, Chi-Mei Medical Center, Liouying, Tainan 73657, Taiwan. This affiliation has been updated.

**Error in Figures**

In the original publication [1], there was a mistake in Figure 1C as published. The β-action 42 kDa in Figure 1C is the same as the MEK 45 kDa in Figure 5A. The corrected Figure 1C appears below. In addition, there was a mistake in Figure 3 as published. The corrected original and magnification images for Zoomed-In Parts are both shown in Figure 3. The corrected Figure 3 appears below. Moreover, higher resolution Figure 4, Figure 5 and Figure 6 are provided in this correction for better elucidation. This correction was approved by the Academic Editor. The original publication has also been updated.

## Figures and Tables

**Figure 1:**
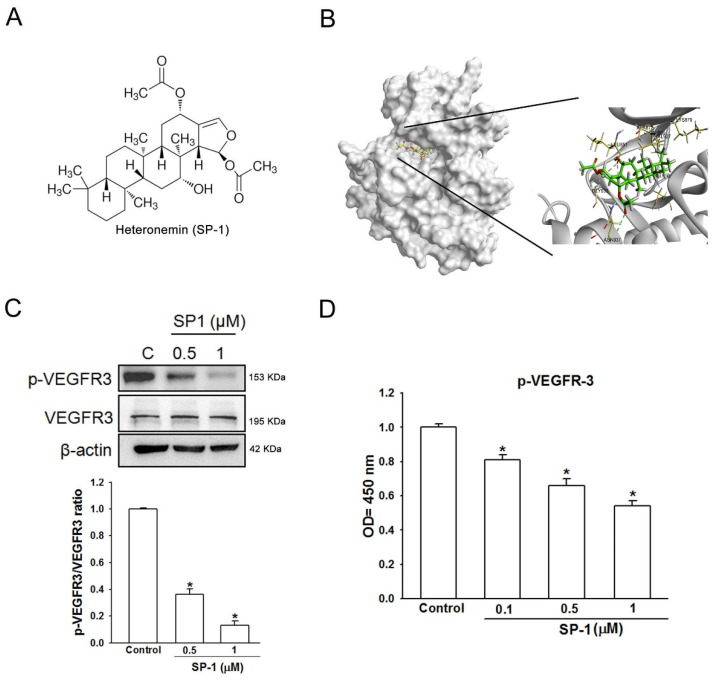
Heteronemin is a VEGFR-3 binding compound in human lymphatic endothelial cells. (**A**) The structure of heteronemin (indicated as SP-1). (**B**) Docking models of SP-1-targeted VEGFR-3. The structure of VEGFR-3 was downloaded from PDB (accession: 4BSJ_A) and represented as gray. SP-1, drawn by ChemDraw Ultra 9.0, rendered in the representation of green stick. Close-up of SP-1 docking site (best energy mode) was prepared using Discovery Studio 4.1. (**C**) Human lymphatic endothelial cells (LECs) were treated with SP-1. Then, the expression of VEGFR-3 and phospho-VEGFR-3 (p-VEGFR-3) was determined by Western blot analysis. The quantitative densitometry of the relative levels of VEGFR-3 and phospho-VEGFR-3 was measured by Image-Pro Plus. (**D**) Cells were stimulated with SP-1; the phospho-VEGFR-3 protein expression in cell lysate was measured by ELISA. A representation experiment is shown as the mean ± S.D. for three wells (* *p* < 0.05). Similar results were observed from three independent experiments.

**Figure 3:**
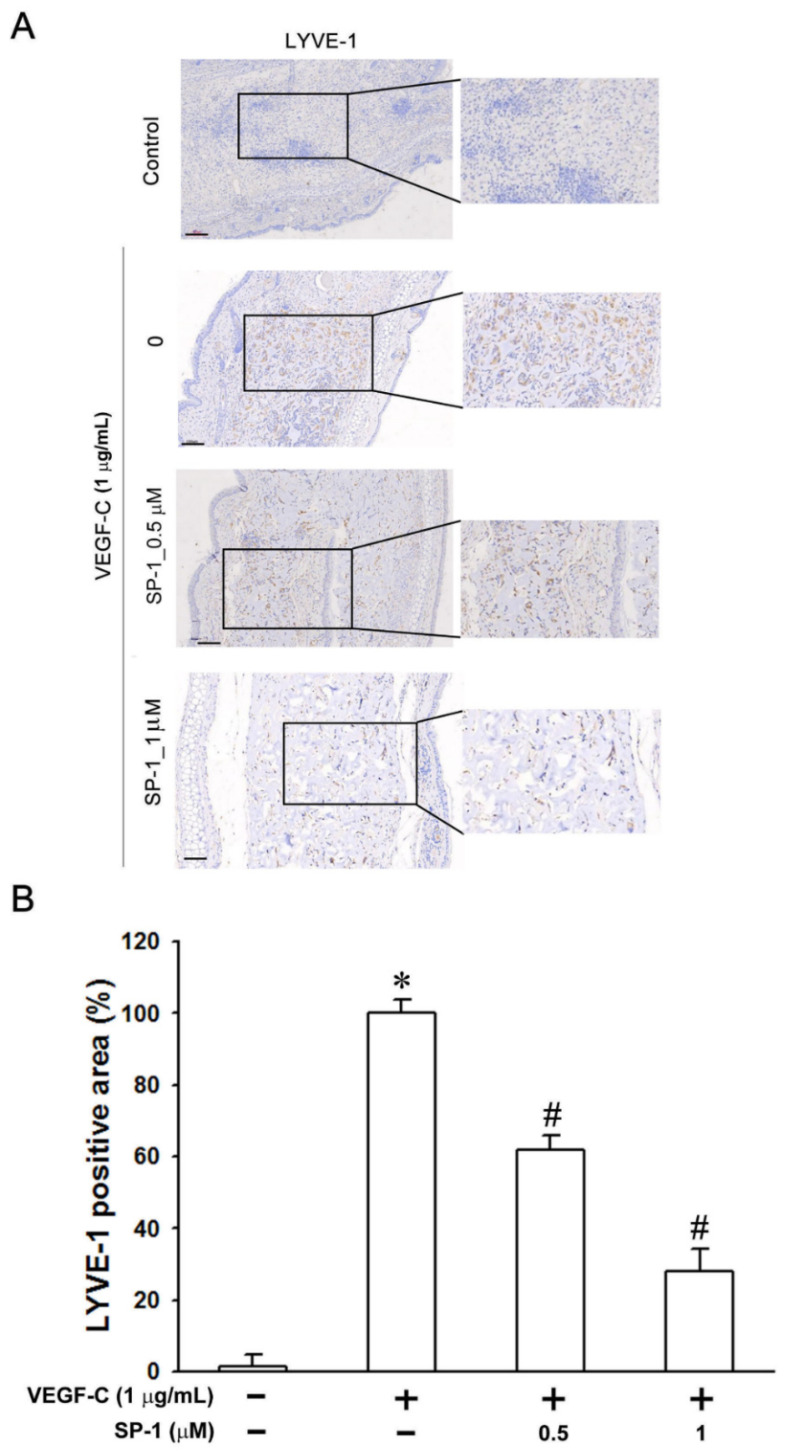
Heteronemin impairs the in vivo VEGF-C-stimulated lymphangiogenesis in mouse ear collagen sponges. Gelatin sponges were soaked with either medium containing VEGF-C (1 μg/mL) as a positive control or VEGF-C combined with the indicated concentration of heteronemin (represent as ‘SP-1’ in the graphs). Sponges were implanted between the two skin layers of mice ears for 3 weeks. (**A**) Lymphatic vasculatures were examined by LYVE-1 immunostainings. (bars = 100 and 70 μmon higher magnification). (**B**) The graphs represent the computerized quantification of the densities of lymphatic vessels, defined as the area occupied by vessels divided by the area of the sponge section. The data are expressed as the means ± SEM of five mice. * *p* < 0.05 versus medium sponges; # *p* < 0.05 versus VEGF-C-stimulated sponges.

**Figure 4:**
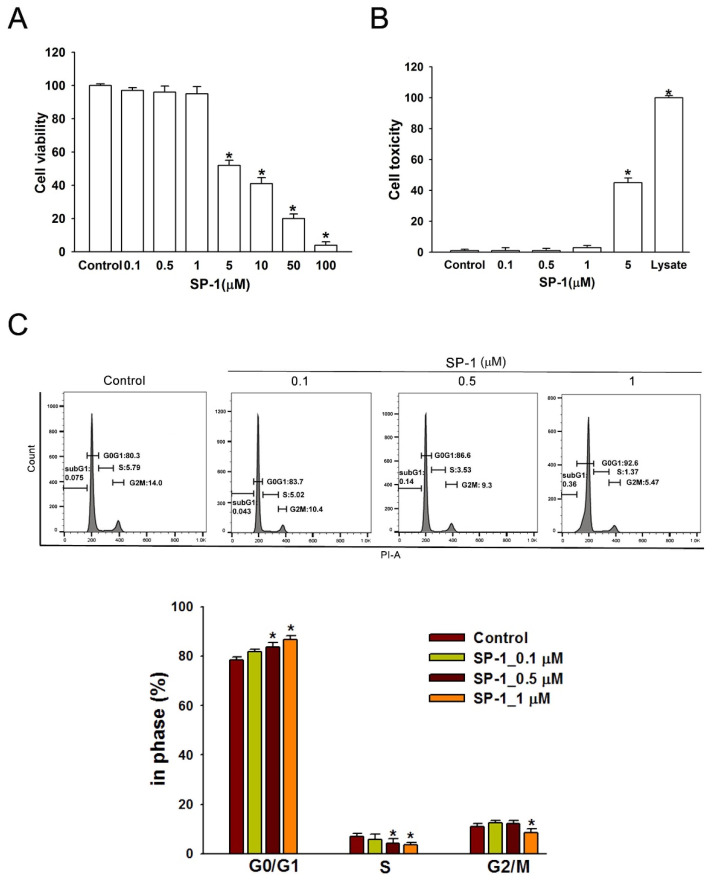
Heteronemin inhibited cell growth and arrested the distribution of cell cycle in human lymphatic endothelial cells. (**A**) LECs were incubated with various doses (0–100 μM) of heteronemin (indicated as SP-1) in a 96-well plate for 24 h. Cell viability was determined by MTT assay after treatment. The maximal non-toxic dose was chosen for further experiments. (**B**) Cells were treated with SP-1 for 8 h; then, the cytotoxicity was determined using LDH assay. (**C**) Cells were treated with various concentrations of SP-1 for 24 h and were then analyzed using flow cytometry. Histograms represent the percentage of cells in each cell cycle phase. The graph corresponds to the distribution of cell subpopulation percentages expressed as means ± SEM of five independent assays. * *p* < 0.05 compared with solvent control (0.01% DMSO).

**Figure 5:**
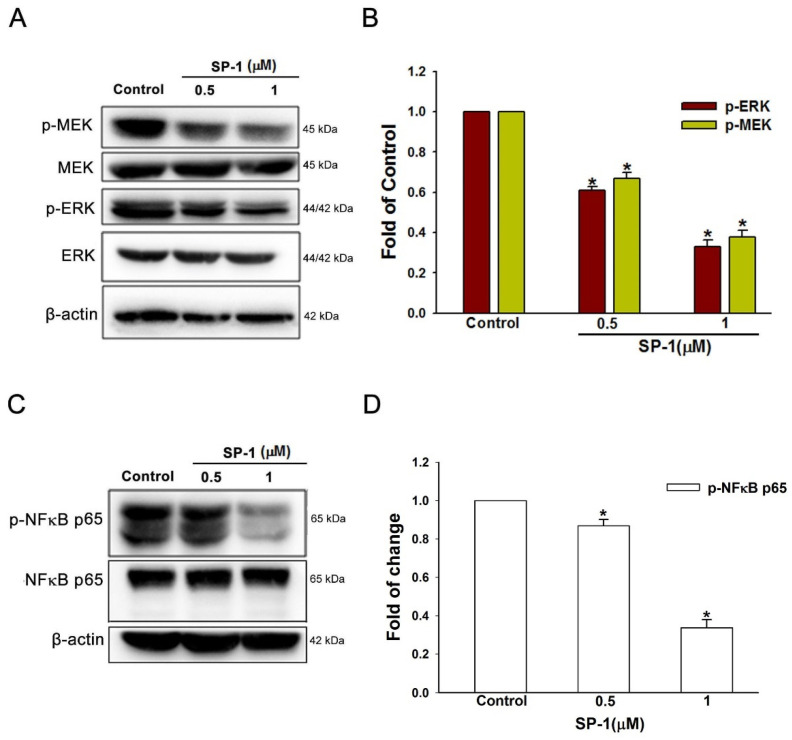
Heteronemin inhibited mitogen-activated protein kinase and transcription factors in human lymphatic endothelial cells. Cells were treated with the indicated concentrations of heteronemin (represented as SP-1); subsequently, the indicated (**A**) MEK/ERK and (**C**) p65 phosphorylated and total proteins were determined by Western blot analysis. The quantitative densitometry of phosphorylated (**B**) MEK/ERK and (**D**) p65 protein was performed with Image-Pro Plus. Data are expressed as mean ± SEM of five independent experiments. * *p* < 0.05, compared with the control group.

**Figure 6:**
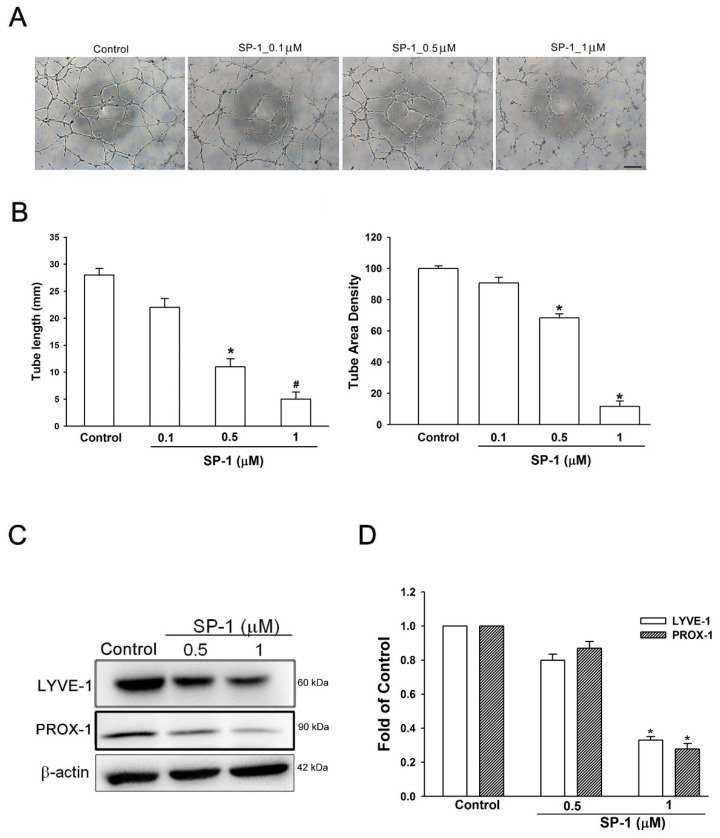
Heteronemin suppressed the tube formation, migration and phosphorylation of VEGF-C/VEGFR-3/LYVE-1 in human lymphatic endothelial cells. (**A**) Cells were treated with the indicated concentrations of heteronemin (indicated as SP-1). The capillary-like structure formation was examined by tube formation (scale bar = 500 μm; 20× magnification). (**B**) The quantification of tube formation was performed using Image-J to validate the anti-lymphangiogenic property of SP-1. (**C**) LECs were treated with SP-1. Then, the expression of LYVE-1and PROX-1 was determined by Western blot analysis. (**D**) The quantitative densitometry of the relative levels of LYVE-1 and PROX-1 was measured by Image-Pro Plus. Data are expressed as the mean ± SEM of at least three independent experiments. * *p* < 0.05; ^#^ *p* < 0.01 compared with the control group.
